# Distinct Aging Effects on Functional Networks in Good and Poor Cognitive Performers

**DOI:** 10.3389/fnagi.2016.00215

**Published:** 2016-09-09

**Authors:** Annie Lee, Mingzhen Tan, Anqi Qiu

**Affiliations:** ^1^Department of Biomedical Engineering, National University of SingaporeSingapore, Singapore; ^2^Clinical Imaging Research Center, National University of SingaporeSingapore, Singapore; ^3^Singapore Institute for Clinical Sciences, the Agency for Science, Technology and ResearchSingapore, Singapore

**Keywords:** brain network hubs, default mode network, central executive network, salience network, intra-extra dimensional set shift, paired associates learning

## Abstract

Brain network hubs are susceptible to normal aging processes and disruptions of their functional connectivity are detrimental to decline in cognitive functions in older adults. However, it remains unclear how the functional connectivity of network hubs cope with cognitive heterogeneity in an aging population. This study utilized cognitive and resting-state functional magnetic resonance imaging data, cluster analysis, and graph network analysis to examine age-related alterations in the network hubs’ functional connectivity of good and poor cognitive performers. Our results revealed that poor cognitive performers showed age-dependent disruptions in the functional connectivity of the right insula and posterior cingulate cortex (PCC), while good cognitive performers showed age-related disruptions in the functional connectivity of the left insula and PCC. Additionally, the left PCC had age-related declines in the functional connectivity with the left medial prefrontal cortex (mPFC) and anterior cingulate cortex (ACC). Most interestingly, good cognitive performers showed age-related declines in the functional connectivity of the left insula and PCC with their right homotopic structures. These results may provide insights of neuronal correlates for understanding individual differences in aging. In particular, our study suggests prominent protection roles of the left insula and PCC and bilateral ACC in good performers.

## Introduction

Normal aging is characterized by a gradual decline in cognitive processes such as executive function, episodic memory, working memory and processing speed (Glisky, 2007; Deary et al., 2009). However, there is substantial heterogeneity in cognitive performances, with some healthy adults showing surprisingly high levels of cognitive function regardless of age while others showing cognitive decline with age (Bennett et al., 2002; Pudas et al., 2013). The existence of little to no decline in age-related cognitive performances has led to a proposal of general cognitive reserve mechanism. The notion holds that heterogeneity in the brain characteristics, such as adaptability of the brain networks, may help some individuals to cope with aging better than others, hence sustaining their cognitive performance (Nyberg et al., 2012). Nevertheless, despite extensive literature on the correlates of neural alterations with age-related cognitive decline (Raz and Rodrigue, 2006; Sala-Llonch et al., 2012), it remains unclear how multiple brain regions work together in order to cope with cognitive heterogeneity in an aging population.

In recent years, resting-state fMRI (rs-fMRI) has become influential in understanding the complex pattern of brain functional organization via the examination of the synchronization of resting-state Blood-Oxygen-Level-Dependent (BOLD) signals among brain regions. The functional connectivity between brain regions reflects the level of inter-regions communication (van den Heuvel and Hulshoff Pol, 2010). A more crucial feature of the brain’s functional organization is the presence of a collection of highly interconnected functional brain network hubs, such as the insula, anterior and posterior cingulate (ACC, PCC), superior frontal (SFC), and medial prefrontal cortex (mPFC) (Buckner et al., 2009). These hubs form the central backbone of communication between different brain regions (van den Heuvel and Sporns, 2013) and play a role in adapting behaviors in response to changes in cognitive demand (Albert et al., 2000; Bassett and Bullmore, 2006). These hubs are also pivotal structures of the core neurocognitive functional networks, such as the default mode network (DMN), central executive network (CEN), and salience network (SN), which are important for higher cognitive functions (Buckner et al., 2008; Sridharan et al., 2008; Power et al., 2013).

Interestingly, recent studies suggest that the network hubs are the first set of structures susceptible to Alzheimer’s disease and psychiatric disorders (Beason-Held et al., 2013; Crossley et al., 2014). It has been hypothesized that such insults tend to concentrate on these network hubs as they have an arguably higher energy consumption as compared to non-hub regions. A higher energy cost could render a greater vulnerability to energy delivery deficits, hence resulting in greater sensitivity to neurodegenerative diseases and psychiatric disorders. Indeed, previous studies have shown that the network hubs have greater metabolic rates and blood flow as compared to non-hubs regions (Buckner et al., 2008; Liang et al., 2013; Dai et al., 2014). Moreover, the network hubs are hallmarks of anatomical abnormalities for neurodegenerative and psychiatric disorders (van den Heuvel and Sporns, 2011; Liang et al., 2013; Tomasi et al., 2013). Furthermore, age-related disruptions of functional connectivity among the hubs were also found to be detrimental to episodic memory and executive function in older adults (Andrews-Hanna et al., 2007; Damoiseaux et al., 2008; Hampson et al., 2010; Wang L. et al., 2010; He et al., 2012; Mevel et al., 2013; Razlighi et al., 2014; Beaty et al., 2015), underscoring the importance of the network hubs in understanding normal aging.

While the studies mentioned above have examined the sensitivity of the network hubs to normal aging, it remains unclear how age-related alteration in the hubs and their intrinsic network architecture elucidate the presence of substantial cognitive heterogeneity in an aging population. This study thus aimed to investigate the age effects on the functional connectivity of the aforementioned network hubs in good and poor cognitive performers. We expected that good and poor cognitive performers engage the hubs differently to cope with aging, suggesting different mechanisms of aging adaptation. For this, we first identified good and poor cognitive performers by employing cluster analysis based on age and education with adjusted cognitive scores. This classification method allowed for adequate identification of adults into homogenous and distinct groups based on several key cognitive performances related to aging. Processing speed, attention and memory cognitive scores were included in this study since they have been consistently reported to be associated with aging (Lee et al., 2013). We then used complex network measures, derived from brain functional network graphs obtained using rs-fMRI, to provide a comprehensive overview of brain hubs and age-related changes in (1) nodal degree and betweenness centrality of the aforementioned hubs, (2) their functional connectivity, and (3) global and local efficiencies of the brain in good and poor cognitive performers.

We hypothesized that age effects on the topological properties of the hubs are distinct in good and poor cognitive performers. In particular, the right insula and PCC could be key nodes to distinguish good and poor cognitive performers as both network hubs have been highlighted as hallmarks of normal aging associated with cognitive decline (Buckner et al., 2008; He et al., 2013). We therefore expected that the age-related deterioration in the functional connectivity of the right insula and PCC may be more prominent in poor cognitive performers, but may not been observed in good cognitive performers.

## Materials and Methods

### Subjects

Two hundred and fourteen healthy Singaporean Chinese volunteers aged 21 to 80 years old were recruited (males: 93; females: 121) for this study. Volunteers with the following conditions were excluded: (1) major illnesses/surgery (heart, brain, kidney, lung surgery); (2) neurological or psychiatric disorders; (3) learning disability or attention deficit; (4) head injury with loss of consciousness; (5) non-removable metal objects on/in the body such as cardiac pacemaker; (8) diabetes or obesity; (9) a Mini-Mental State Examination (MMSE) score of less than 24 (Ng et al., 2007); (10) no evidence of cerebrovascular disease based on T1-, T2-weighted and magnetic resonance agiography (MRA). Of the 214 recruited participants, only 172 who were right handed and completed both functional and structural MRI scans were included in the final analysis. This study was approved by the National University of Singapore Institutional Review Board and all participants provided written informed consent prior to participation.

### Cognitive Tasks

The CANTAB includes language-independent cognitive tests (Luciana and Nelson, 2002) administered on a computer fitted with a touch-sensitive screen and a 2-button response pad. Participants were first screened on two motor and learning tasks to verify the ability to follow simple instructions. Subsequently, participants performed seven CANTAB tasks in this order: Intra-Extra Dimensional Set Shift (IED), Match to Sample Visual Search (MTS), Paired Associates Learning (PAL). Our previous study showed age-related decline in the performance of these cognitive tasks (Lee et al., 2013).

#### Intra/Extra-Dimensional Shift (IED)

The IED task is an adaptation of the Wisconsin card sorting Test. It consists of maintenance of attention and shifting of attention (Robbins et al., 1998). The IED involves a total of nine stages. In each trial participants are shown two abstract images, each comprised of a shape and an overlapping line. They are instructed to choose the correct image in accordance with an underlying rule. For each stage, six continuous correct responses are needed before the task moves to the next stage. If the six continuous correct responses are not obtained within the fifteen trials in each stage, the task is automatically terminated. The number of errors committed on stage 1 indicates proficiency in detecting and learning the implicit rule of the task based on the feedback as to whether the choice is correct. Stage 6 involves an intra-dimensional shift (IDS stage) where shape remains the target cue, but the ‘correct’ shape changes. Stage 8 is the extra-dimensional shift (EDS stage) where participants must learn to shift attention from the previously correct dimension (the shape of the stimulus) to the newly correct dimension (the line). The number of errors made at the IDS stage 6 indicates proficiency in intra-dimensional set-shifting and the number of errors made at EDS stage 8 indicates proficiency in extra-dimensional set-shifting. The total number of errors from stages 1 to 7, referred to as Pre-EDS errors, indicates proficiency in maintaining selective attention.

#### Match-to-Sample Visual Search (MTS)

Match-to-sample visual search (MTS) requires a visual search of a target pattern amongst distracter patterns with a speed-accuracy trade-off. The task begins with a total of eight white empty boxes encircling a red box in the middle of the screen. Participants press the button on the response box to start, after which a target pattern appears in the red box. After a two second delay, either one, two, four, or eight patterns appear in the white boxes. Participants select the pattern that matches that in the red box. In addition, participants are informed not to release their fingers from the response box until they have decided upon the pattern which matches the target. The measure of interest for this task is the MTS reaction time, which is measured from the time the patterns appear to the time the participants release their hands from the response pad.

#### Paired Associates Learning (PAL)

The paired associates learning (PAL) is a visuospatial associative learning task. Participants are presented with six white squares arranged in a circle. Patterns are sequentially presented in each of the six squares for three seconds in a random order. Subsequently, the same patterns are represented in the center of the screen in a different order. The participants are instructed to select the square indicating the original location of the pattern. No time limit is enforced. The number of patterns presented increases if participants correctly locate the original location of every pattern on the first attempt. In the first stage only one pattern is presented, increasing to two patterns in stage 2, three patterns in stage 3, six patterns in stage 4 and finally eight patterns in stage 5. If participants fail to recall the locations correctly, the patterns in that stage are represented for up to 10 attempts per stage. Failure to recall the correct order after 10 attempts results in the termination of the task. The first trial memory score is calculated as the number of objects correctly associated to their locations in the first attempt for each trial in each stage. The first three stages consist of two trials with novel stimuli. Thus, in stage 1 the maximum points that a participant can be awarded are two, followed by four points for forming two correct object-location pairs in stage 2, then six points for forming three correct object-location pairs in stage 3. For stages 4 and 5, each consists of only one (novel) trial, and the maximum number of points awarded is six and eight, respectively. The full score of the first trial memory for the task is 26 points. A higher score indicates better associative learning.

### Cluster Analysis

Prior to cluster analysis, the raw cognitive scores, including Pre-EDS, IDS stage 6, and EDS stage 8 scores, MTS reaction time, and PAL first trial memory scores, were adjusted for age and educational level. This was determined using linear regression with age and educational level as main factors and the residuals were defined as adjusted cognitive scores. For the purpose of cluster analysis, the adjusted cognitive scores were then, respectively, standardized by subtracting the mean and dividing the standard deviation of each measure. The Pre-EDS, IDS stage 6 and EDS stage 8 scores were inversed and referred as Pre-EDS attention, IDS stage 6 attentional flexibility, and EDS stage 8 attentional flexibility, respectively, so that their higher scores indicate better performance.

Cluster analysis on the aforementioned adjusted and standardized cognitive scores was performed using MATLAB R2007b (The Mathworks Inc., Natick, MA, USA). Hierarchical agglomerative clustering was first employed to predefine the number of clusters and estimate an initial cluster center. K-means clustering was then used to determine the cluster membership of each subject. In further assessing stability of cluster memberships, cluster analysis was replicated 1000 times, in which 80% of the subjects were randomly selected. The probability of a subject assigned to the same group across these 1000 trials was computed. Only subjects with this probability greater than 0.80 were included in this study.

### MRI Acquisition

MRI scans were acquired in a 3T Siemens Magnetom Trio Tim scanner using a 32-channel head coil at the Clinical Imaging Research Centre of the National University of Singapore. The image protocols were (i) high-resolution isotropic T_1_-weighted Magnetization Prepared Rapid Gradient Recalled Echo (MPRAGE; 192 slices, 1 mm thickness, sagittal acquisition, field of view 256 mm × 256 mm, matrix = 256 × 256, repetition time = 2300 ms, echo time = 1.90 ms, inversion time = 900 ms, flip angle = 9°); (ii) isotropic axial resting-state functional MRI imaging protocol (single-shot echo-planar imaging; 48 slices with 3 mm slice thickness, no inter-slice gaps, matrix = 64 × 64, field of view = 192 mm × 192 mm, repetition time = 2300 ms, echo time = 25 ms, flip angle = 90°, scanning time = 6 min). During the rs-fMRI scan, the subjects were asked to close their eyes.

### MRI Data Preprocessing

#### Structural MRI Analysis

FreeSurfer ^1^ was used to analyze the T_1_-weighted images and parcellate each hemisphere of the brain into 34 cortical regions and 6 subcortical regions (Fischl et al., 2002, 2004).

#### Rs-fMRI Network Analysis

The rs-fMRI data were first processed with slice timing, motion correction, skull stripping, band-pass filtering (0.01-0.08 Hz) and grand mean scaling of the data (to whole brain modal value of 100). Framewise displacement (head motion characteristics) were computed. The volumes with framewise displacement greater than 0.5 mm and the volumes prior and subsequent to them were removed for the following analysis (Power et al., 2012). The number of volumes removed was up to maximum of 20 and was not correlated with age (*p* > 0.05). Within each subject, the six parameters of the head motion and CSF and white matter signals were regressed out from the fMRI images. Finally, the fMRI images were aligned to the T_1_-weighted image using a boundary based registration algorithm (Greve and Fischl, 2009).

### Functional Network Analysis

**FIGURE 1:**
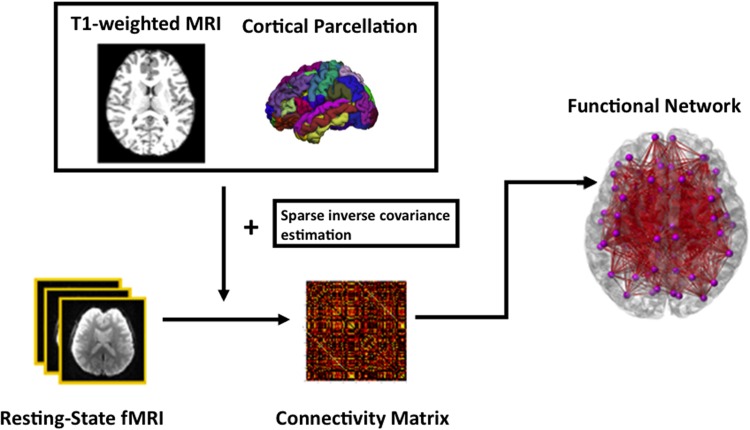
**A schematic diagram of the functional network analysis**.

For functional network analysis, the time series in each ROI parcellated based on FreeSurfer were computed by averaging the signal of all voxels within individual ROIs. Sparse inverse covariance estimation, which provides an estimate of linear conditional dependence between brain regions after removing the linear influence of other brain regions (Ng et al., 2012), takes the times series and then computes a functional connectivity matrix of each subject. Subsequently, a specific sparsity of 0.21 was chosen as threshold at which there is the most significant age difference in the modularity measure. This threshold was chosen in order to capture the most salient age-related changes as proposed in (Wang L. et al., 2010). The data processing of the rs-fMRI is summarized in **Figure 1**.

Subsequently, global efficiency and local efficiency were computed to characterize the potential ease with which information can be transferred concurrently across a network and locally communicated within the neighborhood. At a regional level, betweenness centrality was calculated to identify nodes of a network that play crucial roles in brain network and used to identify hubs. Moreover, nodal degree and connectivity strength were computed to access both topological properties at each node of the SN, DMN and CEN networks as well as processing efficiency (Goh, 2011). Detailed descriptions of these measures are described in (Bullmore and Sporns, 2009; Wang J. et al., 2010; Sporns, 2013) as well below.

#### Global and Local Efficiencies

The *global efficiency* is defined as the mean of the inverse shortest path length in a network (Latora and Marchiori, 2001) and quantifies how efficiently information can be exchanged over the network, considering a parallel system in which each node sends information concurrently along the network. A high global efficiency value may indicate highly parallel information transfer in a network in which each node could efficiently send information concurrently.

In contrast, the local efficiency of a node is calculated as the global efficiency of the neighborhood sub-network of this node, indicating how well the information can be communicated within the neighbors of a given node when this node was removed. The local efficiency across all nodes within a network are further averaged to estimate *the local efficiency of the network* (Rubinov and Sporns, 2010). The local efficiency of a network reflects its potential tendency to present clusters of nodes that deal with common neural information. A high value of the local efficiency of a network may indicate efficiently information transfer in the immediate neighborhood of each node.

#### Betweenness Centrality and Hub

*Betweenness centrality* has been a widely used measure to identify the most central nodes in a network, which act as bridges between the other nodes (Freeman, 1978). The betweenness centrality of a node is defined as the fraction of all shortest paths in the network that pass through the node (Freeman, 1978). A node with high betweenness centrality is thus crucial to efficient communication. Nodes with betweenness centrality more than one standard deviation above the mean of all nodes’ betweenness centrality in the network are defined as hubs (Rubinov and Sporns, 2010).

#### Nodal Degree

*Nodal degree* is calculated as the total number of neighbors of the node. Higher nodal degree ensures an efficient flow of information (Rubinov and Sporns, 2010).

#### Connectivity Strength

*Connectivity strength between two cortical regions* is defined as the edge weight between them. Higher connectivity strength indicates stronger interconnectivity between the two given regions.

### Statistical Analysis

Linear regression was used to examine age effects on nodal degrees, betweenness centralities, and functional connectivity strengths in brain hubs. In a full regression model, linear and quadratic terms of age were entered as main factors and a network metric was entered as dependent variables while controlling for gender (network metric ∼ β_0_ + β_1_ Age + β_2_ Age^2^ + β_3_ Gender + 𝜀). If the quadratic term of age has no significant influence on network measures, a reduced regression model with only the linear term of age as main factor was used (network metrics ∼ β_0_ + β_1_Age + β_2_Gender + 𝜀). These two models were applied to good and poor cognitive performers. Bonferroni correction was carried out for correcting multiple comparisons. Bonferroni corrected significance level was 0.05/12 (total of 12 nodes were used in this study, with 6 nodes (insula, ACC, PCC, mPFC, SFC, and MFC) from each hemisphere) for nodal degree and betweenness centrality and was 0.05/6 (within each of the networks) for intranetwork functional connectivity strength. Simple slope analysis was further employed to compare the slopes of age-related decline in functional connectivity between the good and poor performers.

Partial pearson’s correlation analysis was further used to examine the relationship between cognition and the aforementioned measures of the functional network in good and poor cognitive performers. Note that this analysis was applied only to the network measures significantly associated with age. Statistical analysis was performed using SPSS 18 (SPSS Inc., Chicago, IL, USA) for Windows 7 and MATLAB R2007b (The Mathworks Inc., Natick, MA, USA).

## Results

### Cluster Analysis on Subjects’ Cognitive Performance

**FIGURE 2:**
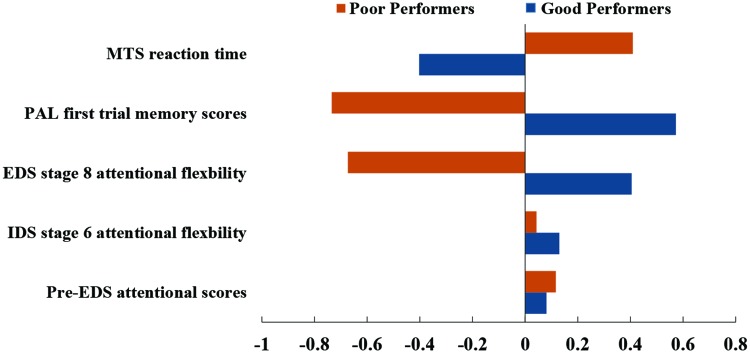
**Cognitive profiles in good cognitive performers (blue) and poor cognitive performers (orange)**.

All 172 subjects completed the MTS, IED, and PAL tasks. Four subjects were excluded from cluster analysis as their cognitive performance scores were three standard deviations below the average cognitive scores. The demographic information of the remaining 168 subjects are listed in **Table 1**. Dendrogram analysis based on the standardized cognitive scores separated the subjects into two groups. The number of subjects in each group was 87 and 81, respectively. **Figure 2** illustrates the bar plot of the adjusted and standardized cognitive scores for each group. After controlling for the educational level and age, the two groups significantly differed in MTS reaction time, extra-dimensional attentional flexibility (EDS stage 8), the first memory ability of PAL (*p* < 0.001) but not in Pre-EDS attention and intra-dimensional attentional flexibility (IDS stage 6) (*p* > 0.05). Henceforth, the subjects in the group with lower MTS reaction time, better extra-dimensional shift ability (EDS stage 8) and the first memory ability of PAL were classified as “good cognitive performers”, while the others were as “poor cognitive performers”.

**Table 1:** Demographic distribution.

Age Range	20s (*n* = 32) mean (*SD*)	30s (*n* = 25) mean (*SD*)	40s (*n* = 26) mean (*SD*)	50s (*n* = 41) mean (*SD*)	>55 (*n* = 44) mean (*SD*)
Female, %	56.3	52.0	61.5	58.5	70.4
Age	25.7 (2.22)	33.6 (2.39)	44.5 (2.79)	54.7 (3.04)	67.4 (4.77)
Education Level	4.59 (0.56)	4.72 (0.54)	3.58 (0.99)	3.12 (1.01)	2.72 (1.45)

*Education Level: 0 = no education, 1 = primary school level, 2 = secondary school level, 3 = Singapore-Cambridge General Certificate of Education Ordinary Level (‘O’ level) / Singapore-Cambridge General Certificate of Education Normal (Academic) Level (‘N’ level), 4 = Pre-University/Diploma/ITE/Certificate, 5 = Degree and above.*

Subsequently, only the PAL first trial memory, EDS stage 8 attentional flexibility, and MTS reaction time scores were used in random sampling and *k*-means classification analysis that identified 155 subjects with the stability of the group membership greater than 0.80. **Table 2** shows the final subjects’ distribution (*N* = 155) of each cluster that were used for further analysis in this study.

**Table 2:** The subject distribution among good and poor cognitive performers in each age decade.

Age Range (*n* = 155)	20s (*n*)	30s (*n*)	40s (*n*)	50s (*n*)	>55 (*n*)
Good Cognitive Performers	20	14	12	7	29
Poor Cognitive Performers	12	9	12	10	30

### Age Effects on Functional Networks in Good and Poor Cognitive Performers

No quadratic effects of age were found on the network measures in both good and poor cognitive performers. Hence, only linear effects of age on the network measures are reported below.

**FIGURE 3:**
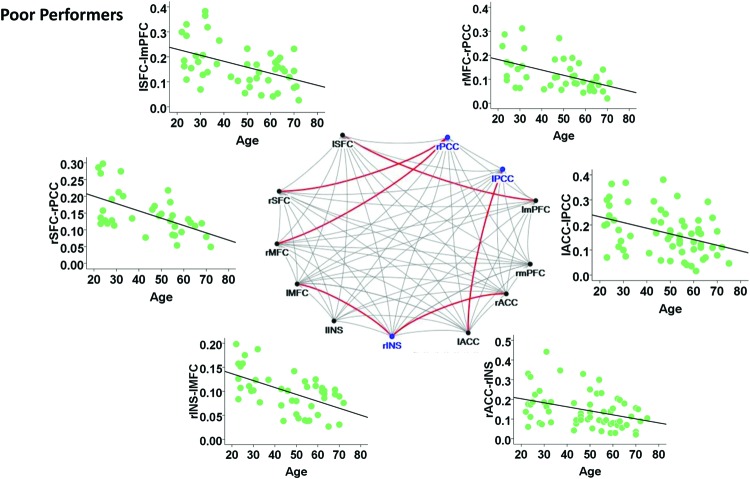
**Internetwork connectivity among the salience network (SN), central executive network (CEN), and default mode network (DMN) in poor cognitive performers.** Blue color balls indicate hubs identified based on betweenness centrality. Red lines indicate significant age-related decreases in functional connectivity strength, where *p* < 0.003 after multiple comparison correction. l, left; r, right; SFC, superior frontal cortex; MFC, middle frontal cortex; INS, insula; ACC, anterior cingulate cortex; mPFC, medial prefrontal cortex; PCC, posterior cingulate cortex.

Among the poor cognitive performers, the right insula and bilateral PCC were identified as hubs, indicating their influential roles in the brain functional network. Our analysis further revealed that numerous age-related alterations in the degrees of these hubs, as well as specific hubs-related functional connectivity strength. Specifically, the poor cognitive performers showed significant age-related decreases in the degrees of the right insula and right PCC (*p* < 0.004; the second column in **Table 3**). Moreover, the right insula also showed age-related decreases in its functional connectivity with the right ACC in SN (β = -0.358, *p* = 0.004) and left middle frontal cortex (β = -0.360, *p* = 0.003) in CEN. The left PCC shows age-related decreases in its functional connectivities with left ACC in the SN (β = -0.396, *p* = 0.002). The right PCC shows age-related decreases in its functional connectivity with the right middle frontal cortex (β = -0.490, *p* < 0.001) and right superior frontal cortex (β = -0.556, *p* < 0.001) in the CEN. The functional connectivity vulnerable to aging is not limited to the hubs’ functional connections. Our results revealed that the functional connectivity between the left superior frontal and left medial prefrontal cortices (β = -0.472, *p* = 0.001) across the DMN and CEN networks were reduced as age increased. **Figure 3** illustrates age effects on these functional connectivities among the SN, DMN, and CEN networks. Coupled with age-related decreases in the functional connectivities with the right insula, the left middle frontal cortex also showed increased betweenness centrality with age (*p* < 0.001; the third column in **Table 3**). In addition to the aforementioned findings on the local topological properties of the three functional networks, the poor performers also showed age-related decreases in global and local efficiencies (*p* < 0.02; **Table 4**).

**Table 3:** Age effects on the nodal degree and betweenness centrality of the key nodes of the salience network (SN), central executive network (CEN), and default mode network (DMN).

	Nodal degree		Betweenness centrality	
	Poor performersβ-value (*p*-value)	Good performersβ-value (*p*-value)	Poor performersβ-value (*p*-value)	Good performersβ-value (*p*-value)
Left ACC	-0.148 (0.215)	-0.320 (0.004)^∗^	-0.074 (0.541)	-0.026 (0.825)
Right ACC	-0.207 (0.081)	-0.349 (0.002)^∗^	-0.082 (0.509)	-0.383 (<0.001)^∗^
Right insula	-0.343 (0.004)^∗^	-0.269 (0.016)	0.121 (0.351)	-0.055 (0.676)
Left insula	-0.227 (0.054)	-0.193 (0.088)	-0.025 (0.833)	-0.063 (0.576)
Left PCC	-0.247 (0.037)	-0.331 (0.006)	0.256 (0.068)	-0.053 (0.692)
Right PCC	-0.389 (<0.001)^∗^	-0.173 (0.122)	-0.026 (0.846)	0.089 (0.481)
Left mPFC	0.168 (0.158)	0.126 (0.265)	0.068 (0.581)	0.182 (0.133)
Right mPFC	0.010 (0.930)	-0.047 (0.778)	0.058 (0.631)	0.189 (0.131)
Left middle frontal	-0.135 (0.257)	-0.063 (0.578)	0.441 (<0.001)^∗^	0.135 (0.244)
Right middle frontal	-0.189 (0.112)	-0.067 (0.553)	0.158 (0.210)	0.108 (0.335)
Left superior frontal	-0.252 (0.033)	-0.272 (0.015)	0.140 (0.251)	-0.200 (0.085)
Right superior frontal	-0.315 (0.007)	-0.310 (0.005)	0.009 (0.941)	-0.154 (0.168)

*Standardized β-values and their corresponding p-values are listed. After Bonferroni correction for multiple comparisons, the significance level for individual statistical tests under each column was chosen as 0.004 (0.05/12). ^∗^p <0.004.*

**Table 4:** Age effects on global and local efficiencies.

Global metrics	Poor performersβ-value (*p*-value)	Good performersβ-value (*p*-value)
Global Efficiency	-0.258 (0.038)^∗^	-0.186 (0.103)
Local Efficiency	-0.255 (0.036)^∗^	-0.218 (0.070)

*Standardized β-values and their corresponding p-values are listed. ^∗^p <0.05.*

**FIGURE 4:**
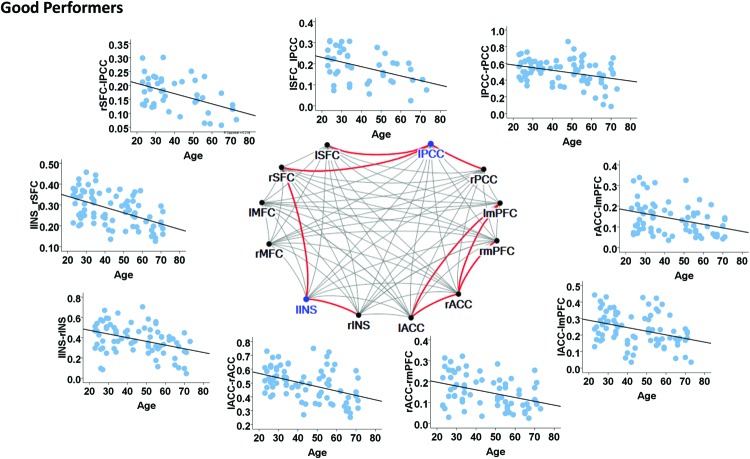
**Internetwork connectivity among the salience network (SN), central executive network (CEN), and default mode network (DMN) in good cognitive performers.** Blue color balls indicate hubs identified based on betweenness centrality. Red lines indicate significant age-related decreases in functional connectivity strength, where *p* < 0.003 after multiple comparison correction. l, left; r, right; SFC, superior frontal cortex; MFC, middle frontal cortex; INS, insula; ACC, anterior cingulate cortex; mPFC, medial prefrontal cortex; PCC, posterior cingulate cortex.

In contrast, the left insula and left PCC were identified as hubs in the good cognitive performers. The good cognitive performers also showed aged-related decreases in hubs’ functional connectivity (**Figure 4**). The left insula had age-related decreases in its functional connectivity with the right insula in the SN (β = -0.390, *p* < 0.001) and the right superior frontal cortex in the CEN (β = -0.528, *p* < 0.001), while the left PCC had age-related decreases in its functional connectivity with the right PCC in the DMN (β = -0.364, *p* = 0.001), the left and right superior frontal cortex in the CEN (left: β = -0.420, *p* = 0.001; right: β = -0.497, *p* = 0.002). Unlike the poor cognitive performers, the good cognitive performers showed the vulnerability of bilateral ACC to aging in terms of their node degree, functional connectivity, and betweenness centrality. The left ACC showed an age-related decrease in its nodal degree (*p* = 0.004; **Table 3**), while the right ACC had age-related decreases in its nodal degree (*p* = 0.002; **Table 3**) and betweenness centrality (*p* < 0.001, **Table 3**). Moreover, the functional connectivity between bilateral ACC within the SN was reduced in aging (β = -0.449, *p* < 0.001). Age-related decreases were also observed in the functional connectivity of bilateral ACC with the left medial prefrontal cortex in the CEN (left: β = -0.391, *p* < 0.001; right: β = -0.373, *p* = 0.002), and between the right ACC and right medial prefrontal cortex in the CEN (β = -0.399, *p* < 0.001). **Figure 4** illustrates these age-related alterations in the functional connectivity. Interestingly, despite the aforementioned age-related alteration seen in the good cognitive performers, both global and local efficiencies were not influenced by aging (**Table 4**).

Slope analysis showed a faster decline in age-related functional connectivity between the right ACC and left medial prefrontal cortices in good cognitive performers as compared to poor cognitive performers (*p* = 0.046). Good cognitive performers also showed a trend of a faster decline in the bilateral ACC functional connectivity as compared to poor cognitive performers (*p* = 0.08). Additionally, poor cognitive performers showed a marginally significant faster decline in the age-related functional connectivity between the right middle frontal cortex and right PCC as compared to the good performers (*p* = 0.061).

### Correlations of the Functional Networks with Cognition

In the good cognitive performers, decreased functional connectivity between left PCC and left superior frontal was associated with better EDS stage 8 scores (*r* = -0.236, *p* = 0.049) after controlling for gender and education level.

In the poor cognitive performers, the functional connectivity strength between the right ACC and right insula (*r* = -0.258, *p* = 0.046) as well as between left PCC and left ACC (*r* = -0.354, *p* = 0.007) correlated with MTS reaction time, suggesting that greater functional connectivity was associated with faster motor speed. In addition, the functional connectivity strength between the left PCC and left ACC was positively correlated with PAL associative learning (*r* = 0.315, *p* = 0.017). Furthermore, the degree of the right PCC was positively associated with associative learning (*r* = 0.275, *p* = 0.021). These findings were obtained after controlling for gender and educational level.

## Discussion And Conclusion

This study utilized cluster and graph network analyses to examine age-related alterations in the functional connectivity of the network hubs in good and poor cognitive performers. Our results emphasized the importance of the insula and PCC in aging in both good and poor cognitive performers. Specifically, poor cognitive performers showed age-dependent disruptions in the functional connectivity of the right insula and PCC. Additionally, poor cognitive performers also showed age-related declines in the left PCC functional connectivities with SN, including the ACC. Conversely, good cognitive performers showed age effects predominantly in the left insula and PCC with the CEN. Interestingly, good cognitive performers showed age-related decreases in the functional connectivities of the left insula and PCC with their right homotopic structures. Our study also underlies the importance of the ACC in the aging processes of good cognitive performers. These results suggest a differential adaptation of hub’s functional connectivity in good and poor cognitive performers as age increases.

Our study identified the insula as a hub in the brain functional network in the whole population (see results of the whole sample in the Supplementary Material), good and poor cognitive performers of this study. Emerging evidence suggests that the insula is an integral hub in assisting target brain regions in the generation of appropriate behavioral responses to salient stimuli (Menon and Uddin, 2010; Laird et al., 2011) as well as involvement in vigilant attention (Langner and Eickhoff, 2013). When such stimuli are detected, the insula facilitates task-related information processing by initializing transient signals that engage brain regions, such as the dlPFC, in order to mediate attentional, working memory, and higher-order cognitive processes while disengaging the structures in DMN (Menon, 2011). Consistent with previous aging studies (Achard and Bullmore, 2007; He et al., 2013, 2014), our results also showed age-related decreases in the functional connectivity of the insula with the dlPFC, and with the ACC in both good and poor cognitive performers. However, we further identified the right insula in poor cognitive performers and the left insula in good cognitive performers as a pivotal structure influenced by aging. Although there are mixed findings on the insula lateralization in structural or functional connectivity (Cauda et al., 2011; Cerliani et al., 2012; Cloutman et al., 2012; Jakab et al., 2012), there are some evidence suggesting that the right insula connects structurally and functionally with more brain regions (Cauda et al., 2011; Cerliani et al., 2012). In addition, the right insula was suggested as a pivotal region for initiating network switching and hence leading to the engagement of the CEN and the disengagement of the DMN. In poor cognitive performers, the right insula reduced its connections with other brain regions in aging. However, its aberrant functional connectivity with the left dlPFC may be compensated via increasing the left dlPFC’s (age-related increase in betweenness centrality of the left dlPFC) ability in communicating with other brain regions. Excessive recruitment of the prefrontal cortex has been consistently shown in fMRI studies on attentional and working memory tasks in the elderly compared to young adults (Reuter-Lorenz et al., 2000; Cabeza et al., 2004; Grady et al., 2008). Taken together, aberrant control signals stemming from the right insula to the CEN may lead to inefficient access to attention and working memory resources, and hence may lead the prefrontal cortex to compensate in normal aging. Furthermore, poor cognitive performers also showed weak connectivity strength of the right insula with the right ACC, which was in turn associated with slower motor speed in older poor cognitive performers. This indicates that functional decoupling between the right insula and ACC may reduce the efficiency of access to the motor system (Laird et al., 2011; Menon, 2011). On the other hand, in good cognitive performers, the left insula reduced its functional connectivity with the right insula as age increased, which may suggest its role in protecting the right insula in aging.

Our study further revealed the PCC as a hub of the brain functional network in the whole population (see the Supplementary Results), good and poor cognitive performers of this study. The PCC is a key node in the DMN (Laird et al., 2009) and is also a highly connected and metabolically active brain region (Buckner et al., 2008; Leech and Sharp, 2014). Similar to the insula, our study identified the right PCC in poor cognitive performers and the left PCC in good cognitive performers as pivotal structures influenced by aging. The right PCC has been found to be involved in spatial orientation (Vogt et al., 2006; Jockwitz et al., 2016). In our study, poor cognitive performers showed that reduced functional connectivity of the right PCC was associated with cognitive decline in associative learning and MTS reaction time. Moreover, a faster rate of decline in the right middle frontal cortex and right PCC functional connectivity in poor cognitive performers as compared to good performers may suggest a detrimental impact on the ability to reorientate attention (Corbetta et al., 2002; Japee et al., 2015). Interaction of the right PCC with other brain regions is also important for attention, working memory, and conscious awareness (Achard and Bullmore, 2007; Bluhm et al., 2008; Damoiseaux et al., 2008; Esposito et al., 2008; Batouli et al., 2009; Koch et al., 2010; Tomasi and Volkow, 2012; Geerligs et al., 2014). Furthermore, the right PCC has also been suggested to reconfigure context-related information, and stand out as transitional hub between the DMN and the right frontal-parietal network during memory recollection (Arenaza-Urquijo et al., 2013), while the left PCC is involved in emotion processing (Colton et al., 2013). Overall, these findings may suggest that the PCC could be an important brain region for understanding heterogeneity of brain and cognitive aging.

Similar to the insula, the PCC also showed an age-related reduction between its interhemispheric connections in good cognitive performers. There is some evidence suggesting that the strength of heterotopic functional connectivities is more predictive of task performances as compared to homotopic functional connectivities. Specifically, a previous study found that greater strength of the heterotopic functional connectivity between different quadrants of visual cortex (i.e., ventral to dorsal) was associated with better perceptual task performances, while no such associations were seen in the homotopic connections (i.e., ventral to ventral) (Bennett et al., 2002; Martin et al., 2012). Together with the absence of age effects on global and local efficiency of the brain functional organization in good cognitive performers, disconnections between homotopic brain regions may indicate a protection role of the left hemisphere to the right hemisphere in good performers.

Of note, our study also revealed the intriguing finding of the ACC with aging in good cognitive performers but not in poor cognitive performers. Age-related decline of glucose metabolism was predominantly found in the medial network involving the ACC and mPFC (Pardo et al., 2007; Vaidya et al., 2007). This decrease in glucose metabolism was correlated with cognitive decline in memory, attention, and verbal fluency (Pardo et al., 2007; Vaidya et al., 2007; Laird et al., 2009). This is in line with our finding of a faster age-associated decline in the functional connectivity between the ACC and the mPFC as compared to poor performers and its correlation with associative learning in good cognitive performers. Furthermore, although both ACC and insula represent the key structures in the SN, the right insula has been suggested to be more vital in triggering control signals to the DMN and CEN networks, partly because it exerts causal influence on the ACC (Sridharan et al., 2008). Despite a faster decline of the ACC homotopic connectivity in good cognitive performers in poor cognitive performers, the right insula could elicit alternative cognitive control mechanism via the CEN and lead to good cognitive performance. This again suggests possible brain pathways to preserve cognitive performance in aging.

The strength of this study was the utilization of cognitive measures and rs-fMRI data, and the employment of cluster analysis for facilitating the exploration of heterogeneity of the brain functional organization in good and poor cognitive performers separately. However, even though the sample size of our study may be adequate, our findings could be biased where majority of the older adults have higher than average MMSE scores as compared to the normal population in Singapore (Ng et al., 2007). Moreover, longitudinal designs might be more appropriate for studying good performance in older adults.

In conclusion, our study provided insights of neuronal correlates in good and poor cognitive performers. In particular, our study suggests the prominent protection roles of the insula and PCC in the left hemisphere, as well as bilateral ACC in good cognitive performers.

## Author Contributions

AL and AQ contributed to study design, data collection, data analysis, and manuscript writing. MT contributed to data analysis.

## Conflict of Interest Statement

The authors declare that the research was conducted in the absence of any commercial or financial relationships that could be construed as a potential conflict of interest.

## Footnotes

1 http://surfer.nmr.mgh.harvard.edu
