# Supplementary material for: Distinct Aging Effects on Functional Networks in Good and Poor Cognitive Performers
Source: Front Aging Neurosci. 2016 Sep 9;8:215. doi: 10.3389/fnagi.2016.00215 (PMC5016512; doi:10.3389/fnagi.2016.00215)
Supplement: Supplementary file 1 [file Presentation_1.PDF]

Supplementary Material

Distinct Aging Effects on Functional Networks in Good and Poor Cognitive Performers

Annie Lee [1], Mingzhen Tan [1], Anqi Qiu[1,2,3*]

Correspondence:

Anqi Qiu PhD

bieqa@nus.edu.sg

Department of Biomedical Engineering, National University of Singapore, 4 Engineering Drive 3, Block E4 #04-08, Singapore 117583.

Results

Age Effects on SN, CEN and DMN networks

Among all the subjects, no quadratic effects of age were found on any functional network metric used in this study. Hence, only linear effects of age on the network measures were reported below.

Bilateral insula and bilateral PCC from the SN and DMN respectively were identified as hubs. Further examination revealed that aging influences not only these hubs but also non-hubs in the three networks of the SN, CEN, and DMN. Age-related reductions in nodal degree were observed in bilateral ACC, PCC, and superior frontal cortex as well as the right insula (**Table S1**). The left middle frontal cortex also showed age-related alterations in betweenness centrality (**Table S1**). Moreover, pertaining to functional connectivity, there was an age-related decrease in intranetwork connectivity strength of the SN and DMN with older adults, suggesting reduced connectivity strength among bilateral insula and ACC as well as

the left PCC with bilateral mPFC and between bilateral PCC (**Table S2**). Significant decreases in internetwork functional connectivity among the three networks were also observed in older subjects. Specifically, the functional connectivities of PCC and mPFC with the CEN and the SN (i.e. superior frontal and left ACC respectively) (**Table S3**) were significantly reduced in older adults.

Table S1. Age effects on SN, CEN and DMN key nodes' degree. Standardized ß-values and their corresponding *p*-values are listed.

	Nodal Degree	*Betweenness Centrality*
	Overall	*Overall*
	ß-value (p-value)	*ß-value (p-value)*
Left ACC	-0.243 (0.003)*	-0.034 (0.684)
Right ACC	-0.292 (<0.001)*	-0.230 (0.007)
Right insula	-0.300 (<0.001)*	0.016 (0.862)
Left insula	-0.222 (0.006)	-0.022 (0.792)
Left PCC	-0.235 (0.003)*	0.107 (0.273)
Right PCC	-0.282 (<0.001)*	0.032 (0.731)
Left mPFC	0.141 (0.082)	0.117 (0.170)
Right mPFC	-0.014 (0.867)	0.125 (0.151)
Left middle frontal	-0.077 (0.343)	0.305 (<0.001)*
Right middle frontal	-0.108 (0.184)	0.126 (0.139)
Left superior frontal	-0.265 (0.001)*	-0.074 (0.380)
Right superior frontal	-0.318 (<0.001)*	-0.073 (0.385)

After Bonferroni correction for multiple comparisons, the significance level for individual statistical tests under each column was chosen as 0.00417 (0.05/12). Hence, $*p < 0.004$.

Table S2. Age effects on functional connectivity strength within SN, CEN and DMN. Standardized ß-values and their corresponding *p*-values are listed.

	Connectivity Strength	*Overall ß-value (p-value)*
Within SN	Right insula-Left insula	-0.337 (<0.001)*
	Right ACC-Left ACC	-0.340 (<0.001)*
	Right insula-Left ACC	-0.225 (0.013)
	Right insula-Right ACC	-0.315 (<0.001)*
	Left insula-Left ACC	-0.219 (0.014)
	Left insula-Right ACC	-0.264 (0.003)*
Within DMN	Left PCC-Right PCC	-0.297 (<0.001)*
	Left mPFC-Right mPFC	0.007 (0.926)
	Left PCC-Left mPFC	-0.257 (0.001)*
	Left PCC-Right mPFC	0.078 (0.340)
	Right PCC-Left mPFC	-0.244(0.002)*
	Right PCC-Right mPFC	-0.085 (0.528)
Within CEN	Left middle frontal-Right middle frontal	-0.041 (0.616)
	Left superior frontal-Right superior frontal	0.020 (0.798)
	Left middle frontal-Left superior frontal	0.119 (0.125)
	Left middle frontal- Right superior frontal	0.163 (0.038)
	Right middle frontal- Left superior frontal	0.111 (0.158)
	Right middle frontal- Right superior frontal	0.109 (0.167)

After Bonferroni correction for multiple comparisons, the significance level for individual statistical tests under each network and per column was chosen as 0.00833 (0.05/6). Hence, *$p < 0.008$.

Table S3. Age effects on functional connectivity strength among SN, CEN and DMN. Standardized ß-values and their corresponding *p*-values are listed.

	Connectivity Strength	*Overall* *ß-value (p-value)*
SN and CEN	Left insula-Left superior frontal	-0.271 (0.047)
	Left insula-Right superior frontal	-0.186 (0.160)
	Left insula-Left middle frontal	-0.111 (0.388)
	Left insula-Right middle frontal	-0.002 (0.991)
	Right insula-Left superior frontal	-0.118 (0.450)
	Right insula-Right superior frontal	-0.209 (0.161)
	Right insula-Left middle frontal	-0.161 (0.073)
	Right insula-Right middle frontal	-0.002 (0.989)
	Left ACC-Left superior frontal	-0.210 (0.012)
	Left ACC-Right superior frontal	-0.077 (0.360)
	Left ACC-Left middle frontal	0.023 (0.886)
	Left ACC-Right middle frontal	0.075(0.402)
	Right ACC-Left superior frontal	-0.171 (0.045)
	Right ACC-Right superior frontal	-0.061 (0.472)
	Right ACC-Left middle frontal	-0.020 (0.823)
	Right ACC-Right middle frontal	-0.024 (0.791)
SN and DMN	Left insula-Left mPFC	0.068 (0.634)
	Left insula-Right mPFC	-0.015 (0.929)
	Left insula-Left PCC	-0.325 (0.004)
	Left insula-Right PCC	-0.088(0.413)
	Right insula-Left mPFC	-0.028(0.884)
	Right insula-Right mPFC	-0.201 (0.256)
	Right insula-Left PCC	-0.321 (0.018)
	Right insula-Right PCC	-0.067 (0.560)
	Left ACC-Left mPFC	-0.268 (<0.001)*
	Left ACC-Right mPFC	-0.173 (0.043)
	Left ACC-Left PCC	-0.353 (<0.001)*
	Left ACC-Right PCC	-0.277 (0.002)*

	Right ACC-Left mPFC	-0.241 (0.005)
	Right ACC-Right mPFC	-0.328 (<0.001)*
	Right ACC-Left PCC	-0.254 (0.005)
	Right ACC-Right PCC	-0.218 (0.017)

After Bonferroni correction for multiple comparisons, the significance level for individual statistical tests under each network and per column was chosen as 0.00313 (0.05/16). Hence, *$p < 0.003$.

Table S3. *(Continue)*

	Connectivity Strength	*Overall* *ß-value (p-value)*
CEN and DMN	Left middle frontal-Left mPFC	-0.091 (0.393)
	Left middle frontal-Right mPFC	-0.003 (0.976)
	Left middle frontal- Left PCC	-0.174 (0.095)
	Left middle frontal- Right PCC	-0.350 (0.002)*
	Right middle frontal-Left mPFC	-0.098 (0.382)
	Right middle frontal-Right mPFC	-0.192 (0.089)
	Right middle frontal- Left PCC	-0.241 (0.027)
	Right middle frontal- Right PCC	-0.211 (0.040)
	Left superior frontal-Left mPFC	-0.344 (0.001)*
	Left superior frontal -Right mPFC	-0.303 (0.004)
	Left superior frontal - Left PCC	-0.251 (0.026)
	Left superior frontal - Right PCC	-0.296 (0.009)
	Right superior frontal -Left mPFC	-0.242 (0.022)
	Right superior frontal-Right mPFC	-0.269 (0.061)
	Right superior frontal - Left PCC	-0.228 (0.039)
	Right superior frontal - Right PCC	-0.332 (0.002)*

After Bonferroni correction for multiple comparisons, the significance level for individual statistical tests under each network and per column was chosen as 0.00313 (0.05/16). Hence, *$p < 0.003$.
